# Sensitivity of Pancreatic Cancer Cell Lines to Clinically Approved FAK Inhibitors: Enhanced Cytotoxicity Through Combination with Oncolytic Coxsackievirus B3

**DOI:** 10.3390/ijms26146877

**Published:** 2025-07-17

**Authors:** Anja Geisler, Babette Dieringer, Leslie Elsner, Maxim Girod, Sophie Van Linthout, Jens Kurreck, Henry Fechner

**Affiliations:** 1 Department of Applied Biochemistry, Institute of Biotechnology, Technische Universität Berlin, 10623 Berlin, Germany; 2 BIH Center for Regenerative Therapies (BCRT), Berlin Institute of Health (BIH), at Charité-Universitätsmedizin Berlin, 13353 Berlin, Germany

**Keywords:** pancreatic cancer, pancreatic ductal adenocarcinoma, focal adhesion kinase, focal adhesion kinase inhibitor, oncolytic coxsackievirus, Chou–Talalay method

## Abstract

Pancreatic ductal adenocarcinoma (PDAC) is a highly aggressive cancer characterized by a dense desmoplastic stroma and a highly immunosuppressive tumor microenvironment (TME). The focal adhesion kinase (FAK), a non-receptor tyrosine kinase, is considered a critical regulator of various cellular processes involved in cancer development. FAK inhibitors (FAKi) have proven to be promising therapeutics for cancer treatment including for pancreatic cancer. As monotherapy, however, FAKi showed only a modest effect in clinical studies. In this study, we investigated the cytotoxicity of six FAKi (Defactinib, CEP-37440, VS-4718, VS-6062, Ifebemtinib and GSK2256098) used in clinical trials on five pancreatic tumor cell lines. We further examined whether their anti-tumor activity can be enhanced by combination with the oncolytic coxsackievirus B3 (CVB3) strain PD-H. IC_50_ analyses identified Defactinib and CEP-37440 as the most potent inhibitors of tumor cell growth. VS-4718, VS-6062, and Ifebemtinib showed slightly lower activity, while GSK2256098 was largely ineffective. The combination of Defactinib, CEP-37440, VS-4718, and VS-6062 with PD-H resulted in varying effects on cytotoxicity, depending on the cell line and the specific FAKi, ranging from no enhancement to a pronounced increase. Using the Chou–Talalay method, we determined combination indices (CI), revealing synergistic, additive, but also antagonistic interactions between the respective FAKi and PD-H. Considering both oncolytic efficacy and the CI, the greatest enhancement in oncolytic activity was achieved when VS-4718 or CEP-37440 was combined with PD-H. These findings indicate that co-treatment with PD-H can potentiate the therapeutic activity of the selected FAKi and may represent a novel strategy to improve treatment outcomes in PDAC.

## 1. Introduction

Pancreatic cancer is one of the most aggressive and deadliest cancers worldwide. Its incidence has drastically increased during the last three decades from about 200,000 cases per year in 1990 to more than 530,000 cases in 2019 [1]. Currently, only eight out of one hundred patients diagnosed with pancreatic cancer survive more than 5 years [2]. PDAC accounts for 85% of all pancreatic malignancies, making it the most common pancreatic malignancy. In the initial phase of tumor development, mutations in the KRAS gene, which are present in approximately 90% of PDACs and are considered oncogenic driver mutations [3], are the main contributors to the activation of oncogenic signaling cascades and the development of pancreatic intraepithelial neoplasia (PanIN). Acquisition of further mutations, mainly occurring in the p53, CDKN2A, SMAD4, CDK27, p16, BRCA1, and BRCA2 genes [3,4], lead to general oncogenic activity associated with unrestrained proliferation of tumor cells, invasive tumor growth, metastasis, and development of chronic inflammation. Distinctive features of full-blown PDAC are an exceedingly dense stroma that permeates and surrounds the tumor cells, far exceeding what is found in other tumor types [5,6,7] and a highly immunosuppressive TME [8]. Both features contribute to further proliferation of pancreatic tumor cells and represent significant barriers, determining the resistance of PDAC to current therapeutic regimes, including chemo-, radio-, target- and immunotherapies [9,10,11].

The FAK, also known as protein tyrosine kinase 2 (PTK2), is a widely expressed nonreceptor tyrosine kinase [12] that is a key component of the focal adhesion complex, which plays an important role in integrin and growth factor receptor signaling in both normal and cancer cells [13]. Overexpression and/or activation of FAK is present in a variety of human malignancies [12], where it plays a key role in activating proliferation-promoting signals. These include migration, angiogenesis, invasion, and survival of cancer cells as well as tumor stem cell renewal and induction of chemotherapy resistance [13,14]. In addition, FAK appears to play a direct role in tumor metabolism by affecting glucose consumption, lipogenesis, and glutamine dependence [15]. Regarding PDAC, another function of FAK stands out as it is considered a key driver of fibrotic and immunosuppressive TME [16]. The tyrosine (Tyr) phosphorylation site Tyr-397 plays a pivotal role in FAK activation, as its autophosphorylation is a critical step in initiating FAK signaling [17]. Given the important role of FAK in tumor biology, various small molecule FAKi have been developed [18]. Most of these FAKi inhibit the kinase activity of the FAK through targeting of autophosphorylation of Tyr-397 [19]. Preclinical studies have demonstrated the efficacy of the FAKi as monotherapy in pancreatic cancer, leading to reduced tumor growth and an increase in animal survival [16,20]. However, in clinical trials FAKi monotherapies demonstrate only limited efficacy, reflected in modest clinical activity characterized by low rates of partial responses and disease stabilization in a small subset of patients with advanced solid malignancies, such as colorectal, ovarian, lung, and pancreatic cancers [21,22,23,24,25]. Recent studies have demonstrated improved anti-tumor effects in preclinical settings by combining FAKi with immune checkpoint inhibitors [26], chemotherapy [18,27], radiotherapy [28], virotherapy [29,30], targeted agents [31] and in clinical settings by combining FAKi with checkpoint inhibitors and chemotherapy [32] resulting in a resistance overcoming, enhanced immune response, and improved overall anti-tumor effect in pancreatic cancer.

Oncolytic viruses (OV) selectively infect and kill tumor cells and induce a strong systemic anti-tumor immune response [33]. Their therapeutic efficacy has been demonstrated across various cancer types in both preclinical and clinical studies. To date, four OV have been approved for clinical use: T-VEC and G47Δ [34,35], both genetically modified herpes simplex virus type 1 strains, for malignant melanoma and glioblastoma, respectively; and the engineered adenoviruses rAd-p53 and H101 [36,37], for the treatment of head and neck cancer. We have developed the OV PD-H, a single-stranded RNA virus belonging to the CVB3 group [38]. The virus exhibits a unique receptor tropism for N- and 6-*O*-sulfated heparan sulfates, which determines its tumor cell tropism and distinguishes it from other CVB3 strains [39]. PD-H efficiently infects and lyses colorectal and pancreatic tumor cells in vitro and suppresses the growth of colorectal and pancreatic tumors in vivo [38,39,40]. In combination with chemotherapeutic agents of the FOLFOXIRI regime, it acts synergistically, leading to distinctly stronger destruction of colorectal tumor cells compared to treatment with chemotherapeutic agents or PD-H alone [41].

In this study, we investigated the oncolytic activity of six FAKi currently being evaluated in clinical trials, using various pancreatic tumor cell lines and examined whether tumor cell killing can be enhanced through combination with PD-H. We show that all FAKi killed pancreatic cancer cells but differed in their cytotoxic activity. Co-treatment of pancreatic cancer cells with FAKi and PD-H resulted in a synergistic and additive interaction, leading to enhanced tumor cell lysis compared to treatment with either agent alone. However, antagonistic effects and a lack of enhancement of cytotoxicity in the combination approach were also seen.

## 2. Results

### 2.1. FAKi Induce Dephosphorylation at Tyr-397 of FAK in Pancreatic Tumor Cells

Several FAKi have been evaluated in clinical trials in Phase I to Phase III for the treatment of various types of cancer, including pancreatic cancer. Among these are VS-4718, Ifebemtinib, VS-6062, GSK2256098, CEP-37440, and Defactinib, which are being investigated in this study (Table 1).

**Figure 1:**
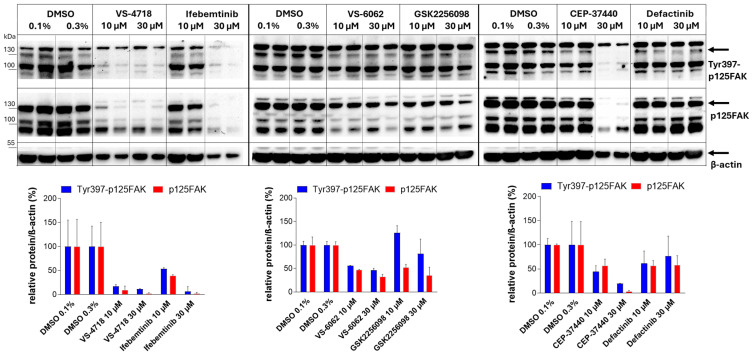
Detection of Tyr-397 phosphorylated FAK and total FAK levels in Beta-TC-3 cells following treatment with FAKi. Upper panels: Beta-TC-3 cells were treated with 10 µM or 30 µM of the indicated FAKi (VS-4718, Ifebemtinib, VS-6062, GSK2256098, CEP-37440, or Defactinib) or with DMSO (0.1% or 0.3%) as a vehicle control. After 24 h, protein lysates were analyzed by Western blotting for Tyr-397 phosphorylated FAK (Tyr397-p125FAK) and total FAK (p125FAK). β-actin was used as an internal loading control. Arrows indicate bands corresponding to the expected molecular weight of 125 kDa. Lower panels: Quantification of the indicated protein levels was performed by densitometric analysis using ImageJ version 1.53a software. Expression levels of phosphorylated Tyr-397 FAK and total FAK were normalized to β-actin and are shown relative to DMSO-treated control cells, which were set to 100%. Shown are mean values ± SD from 2 samples. Notable both phosphorylated and total FAK proteins were not only detected at the expected molecular weight of 125 kDa but also at higher and lower molecular weights, suggesting the presence of multiple FAK isoforms in Beta-TC-3 cells [42,43].

These FAKi inhibit the autophosphorylation of FAK at Tyr-397 and thereby block its catalytic kinase activity [19]. To confirm the functionality of the FAKi and to show that they exert their effect in pancreatic cancer cells, the pancreatic tumor cell line Beta-TC-3 was treated with 10 µM or 30 µM of the respective FAKi. Proteins were isolated from the cells 24 h post-treatment, and the levels of Tyr-397 phosphorylated FAK, total FAK, and β-actin (as a loading control) were analyzed by Western blotting and compared to DMSO-treated cells. Dephosphorylation of Tyr-397-phosphorylated FAK was observed when cells were treated with 10 µM FAKi and more markedly when treated with 30 µM FAKi, with VS-4718, Ifebemtinib, and CEP-37440 being the most potent inhibitors. In contrast, VS-6062 and Defactinib showed moderate effects, while GSK2256098 exhibited minimal activity. The reduction in Tyr-397 phosphorylation was accompanied by a downregulation of total FAK protein levels (Figure 1).

These findings confirm that all tested FAKi are capable of inhibiting Tyr-397 phosphorylation in pancreatic tumor cells. Furthermore, our results show that there are differences between the FAKi with regard to the extent of dephosphorylation.

### 2.2. FAKi Have Different Oncolytic Activity in Pancreatic Tumor Cells

**Figure 2:**
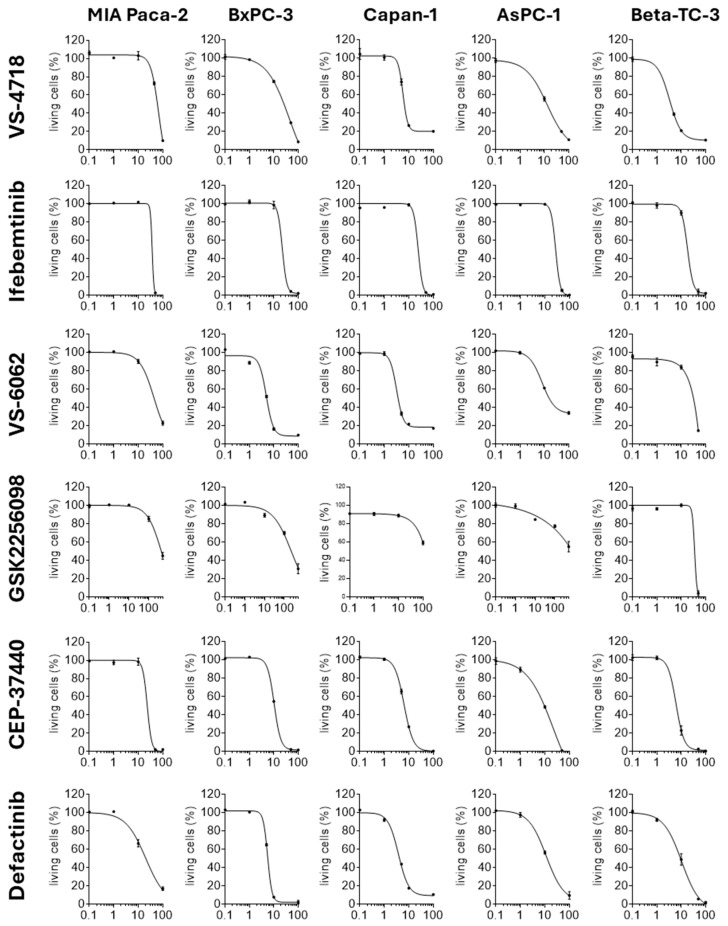
Cytotoxic activity of FAKi with dose–response curves in pancreatic tumor cells. The five pancreatic tumor cell lines MIA Paca-2, BxPC-3, Capan-1, AsPC-1, and Beta-TC-3 were incubated with FAKi VS-4718, Ifebemtinib, VS-6062, GSK2256098, CEP-37440, or Defactinib at concentrations of 0.1–500 µM. Cell viability was determined by XTT assay 72 h post-treatment. Data were set relative to DMSO-treated cells. Shown are the nonlinear fits of each FAKi with mean values ± SD from 3 independent experiments.

To elucidate the oncolytic effects induced by the FAKi in pancreatic tumor cells, we carried out dose-response investigation to determine the cytotoxicity and IC_50_ for each FAKi on five pancreatic tumor cell lines (MIA Paca-2, BxPC-3, Capan-1, AsPC-1, and Beta-TC-3). The tumor cell lines were treated with FAKi concentrations ranging from 0.1 to 500 µM and cytotoxicity was assessed 72 h post-treatment using the XTT assay (Figure 2). At concentrations of 0.1 and 1 µM, investigated FAKi induced little to no cytotoxicity in the cell lines. At a higher concentration of 10 µM, cytotoxic effects became evident; however, differences were observed in both the extent of cytotoxicity induced by the individual FAKi and the sensitivity of the respective cell lines to the FAKi. VS-4718, VS-6062, CEP-37440, and Defactinib mainly exhibited moderate to high cytotoxicity in the majority of cell lines tested. In contrast, Ifebemtinib did not induce cytotoxic effects at a concentration of 10 µM. A similar trend was observed for GSK2256098, which exhibited no cytotoxicity in three cell lines (MIA Paca-2, Capan-1, and Beta-TC-3), while only minimal cytotoxic effects were observed in BxPC-3 and AsPC-1 at the same concentration. Higher concentrations of 100 µM enhanced the cytotoxicity of the FAKi. Defactinib, CEP-37440, and Ifebemtinib were most active at this concentration, resulting in near-complete lysis of almost all cell lines. Interestingly, Ifebemtinib showed the most distinct and unique dose–response curve among all tested FAKi, with a dramatic increase in cell toxicity observed between 10 and 100 µM for all cell lines. Slightly lower maximal efficacy was observed for VS-4718 and VS-6062, with 10–20% and 10–40% of cells remaining viable, respectively. GSK2256098 showed the lowest cytotoxic potency. Using it, 60–90% of cells remained viable in four out of five tested cell lines when 100 µM of the drug was used. Even when the concentration of GSK2256098 was increased to 0.5 mM in MIA Paca-2, BxPC-3, and AsPC-1, the cytotoxicity was only moderately further increased, with 30–60% of tumor cells still viable. Utilizing the dose–response relationships, the IC_50_ values were calculated for each FAKi for the different cell lines and subsequently an average IC_50_ value was calculated for each compound in all pancreatic tumor cell lines (Table 2). According to these results, Defactinib and CEP-37440 had the lowest IC_50_ values, at 10.35 and 11.23 µM, respectively. In contrast, the IC_50_ values for VS-6062, VS-4718, and Ifebemtinib were approximately twice as high, at 20.07, 21.87, and 25.95 µM, respectively. Notably, the IC_50_ for GSK2256098 was distinctly higher, at 308.97 µM. Regarding the sensitivity of the pancreatic tumor cell lines to the respective FAKi, the calculated IC_50_ values indicated that the MIA Paca-2 cell line exhibited low sensitivity, BxPC3 and AsPC-1 displayed moderate sensitivity, and Capan-1 and Beta-TC-3 showed marked sensitivity (Figure 2).

In conclusion, these data demonstrate that all FAKi induce cytotoxicity in pancreatic tumor cells. However, the cytotoxic effects of the FAKi varied significantly and were dependent on the cell line examined. The most effective FAKi were CEP-37440 and Defactinib, whereas GSK2256098 was the FAKi with the lowest cytotoxic efficiency.

### 2.3. Pancreatic Tumor Cells Exhibit Different Sensitivity to the Oncolytic CVB3 PD-H

**Figure 3:**
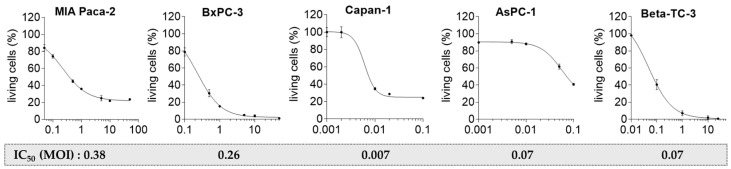
Oncolytic activity of PD-H with dose–response curves in pancreatic tumor cells. The indicated pancreatic tumor cell lines were infected with PD-H at 0.001 to 50 MOI, and cell viability was determined 72 h post-infection by XTT assay, except for Beta-TC-3 cells in which cell viability was determined 48 h post-infection. Data were set relative to untreated cells. Shown are the nonlinear fits of PD-H with mean values ± SD from 3 independent experiments. IC_50_ values of PD-H were calculated from measurements and indicated below each diagram.

To assess the sensitivity of the five pancreatic tumor cell lines to PD-H, the cells were infected with 0.001 to 50 MULTIPLICITY OF INFECTION (MOI) of the virus, and the cytotoxicity was assessed by XTT assay 72 h later. MIA Paca-2 cells were highly resistant to PD-H. When treated with 1 MOI of PD-H, about 36% of the cells remained viable. Even when the dose was increased to 50 MOI, the amount of viable cells decreased only moderately to approximately 20%. The cell line BxPC-3 was more sensitive. At 1 MOI, less than 20% were still alive, and nearly complete cell lysis was observed at a PD-H dose of 50 MOI. Capan-1, AsPC-1, and Beta-TC-3 were the most sensitive cell lines. At 0.1 MOI, only 20–40% of the cells were alive. We also calculated the IC_50_ values for PD-H, which varied significantly across the cell lines analyzed (range: 0.38 MOI in MIA Paca-2 to 0.007 MOI in Capan-1) (Figure 3). These data demonstrate a differential sensitivity of the pancreatic tumor cell lines to PD-H.

### 2.4. The Combination of FAKi and PD-H Can Induce a Higher Cytotoxicity in Pancreatic Tumor Cells than Treatment with Either Agent Alone

**Figure 4:**
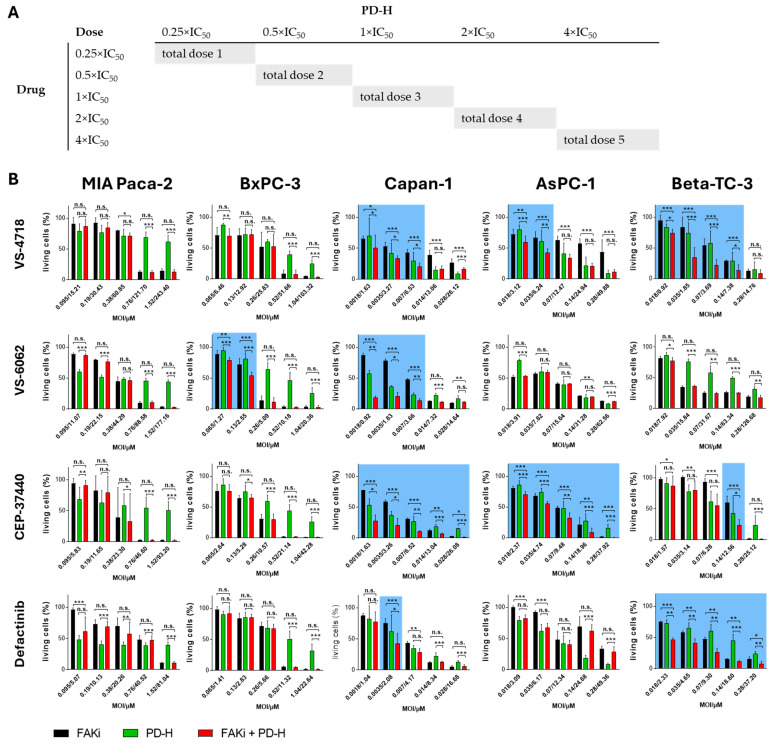
Cell growth inhibition upon combination of FAKi with PD-H. (**A**) Application scheme. Shown is the diagonal constant ratio scheme and the resulting data points used for measurement. (**B**) Cell growth inhibition. The pancreatic tumor cells were infected with PD-H for 1 h and treated thereafter with FAKi or the cells were treated with PD-H or the indicated FAKi drugs alone. Cell viability was measured by XTT assay 72 h or 48 h (only Beta-TC-3) post-treatment. Shown are mean values ± SD from minimum 3 independent experiments. Statistical significance, * *p* < 0.05, ** *p* < 0.01, and *** *p* < 0.001; n.s., not significant. Highlighted in blue: the combination of FAKi and PD-H resulted in a statistically significantly higher inhibition of tumor cell growth compared to both FAKi and PD-H alone.

To determine whether and how PD-H complements the oncolysis of the FAKi, we employed the diagonal constant ratio design proposed by Chou and Talalay [44]. This approach not only allows the determination of whether the combination of FAKi and PD-H results in increased cytotoxicity compared to either agent alone but also enables the calculation of a CI, which helps to assess whether the two agents interact synergistically, additively, or antagonistically. For these investigations, we included only four of the six FAKi, VS-4718, VS-6062, CEP-37440 and Defactinib, as the remaining FAKi exhibited substantially higher IC_50_ values for cell cytotoxicity (Table 2). For the analysis, a diagonal constant ratio scheme was developed based on the IC_50_ values determined for both the FAKi and PD-H (Figure 4A). Each pancreatic tumor cell line was treated either with the FAKi and PD-H alone or in combination according to the diagonal constant ratio scheme. The inhibition of cell growth was determined relative to control cells by XTT assay after 72 h or after 48 h for Beta-TC-3 cells.

The combination of VS-4718 and PD-H resulted in a significant increase in cytotoxicity in Capan-1, AsPC-1, and Beta-TC-3 cells compared to treatment with either FAKi or PD-H alone. However, the effect was restricted to low and medium doses of PD-H and VS-4718. In contrast, in MIA Paca-2 and BxPC-3 cells, no increase in the cytotoxicity of the combination approach was observed across the entire dose spectrum. Similar results were observed for the combination of CEP-37440 and PD-H. The cell lines MIA Paca-2 and BxPC-3 did not exhibit enhanced cytotoxicity upon combined treatment, whereas significantly increased cytotoxicity was observed in Capan-1, AsPC-1, and Beta-TC-3 cells. However, compared to VS-4718, the enhanced cytotoxicity of the combination approach was detectable over the entire dose range in Capan-1 and AsPC-1 cells, whereas for Beta-TC-3 cells it was evident only at a single data point at medium doses. Treatment with VS-6062 and PD-H or Defactinib and PD-H in combination led to a significantly stronger induction of cytotoxicity in only two of the five cell lines tested compared to the single agents: in BxPC-3 and Capan-1 cells, and in Capan-1 and Beta-TC-3 cells, respectively. For the combination of VS-6062 and PD-H, similar to VS-4718, an increased cytotoxicity of the combination approach was only detected when low or medium doses of the FAKi and the virus were used. In contrast, the combination of Defactinib and PD-H resulted in a significant increase in cytotoxicity across the entire dose range in Beta-TC-3 cells, whereas in Capan-1 cells this effect was limited to a single data point at low doses (Figure 4).

**Figure 5:**
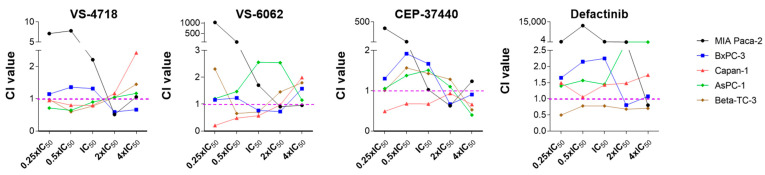
Combination index. The CI value for each measured data point is shown for each combination. CI values below the threshold of 1 indicate synergic, values of about 1 indicate additive, and values above 1 indicate antagonistic interactions between FAKi and PD-H.

Based on the cytotoxicity data obtained in the FAKi/PD-H combination approach, the CI values were calculated for each data point. A CI > 1 indicates antagonism between the two compounds, a CI = 1 suggests additivity, and a CI < 1 indicates synergism. For the combination of VS-4718 and PD-H, additive or synergistic effects were observed at low to medium doses in Capan-1, AsPC-1, and Beta-TC-3 cells, and at medium to high doses in MIA Paca-2 and BxPC-3 cells. In contrast, an antagonistic interaction was observed at high doses in Capan-1 and Beta-TC-3 and at medium and low doses in MIA Paca-2 and BxPC-3 cells. The effects of the combination of VS-6062 and PD-H in MIA Paca-2, BxPC-3, and Capan-1 cells were similar to those observed with the VS-4718/PD-H combination. In contrast, in AsPC-1 cells, VS-6062 and PD-H exhibited only antagonistic interactions across the entire dose range, whereas in Beta-TC-3 cells, antagonistic effects were observed at both low and high doses, with synergistic effects occurring at medium doses. The combination of CEP-37440 and PD-H showed synergistic interactions across the entire dose range in Capan-1 cells, whereas in the other cell lines, synergistic or additive effects were mainly observed at high doses and antagonistic interactions at lower doses. For Defactinib, a synergistic interaction with PD-H was observed across the entire dose range only in Beta-TC-3 cells, while in the other cell lines, antagonistic interactions were more commonly detected (Figure 5).

In conclusion, these data suggest that the combination of FAKi with PD-H can enhance cytotoxic effects compared to treatment with FAKi or PD-H alone. Furthermore, the CI values show that the interaction between FAKi and PD-H can be synergistic, additive, or antagonistic. However, the specific effects vary depending on the FAKi applied, the pancreatic cancer cell line, and the respective doses of FAKi and PD-H.

### 2.5. Treatment of Pancreatic Tumor Cells with FAKi Inhibits PD-H Replication

**Figure 6:**
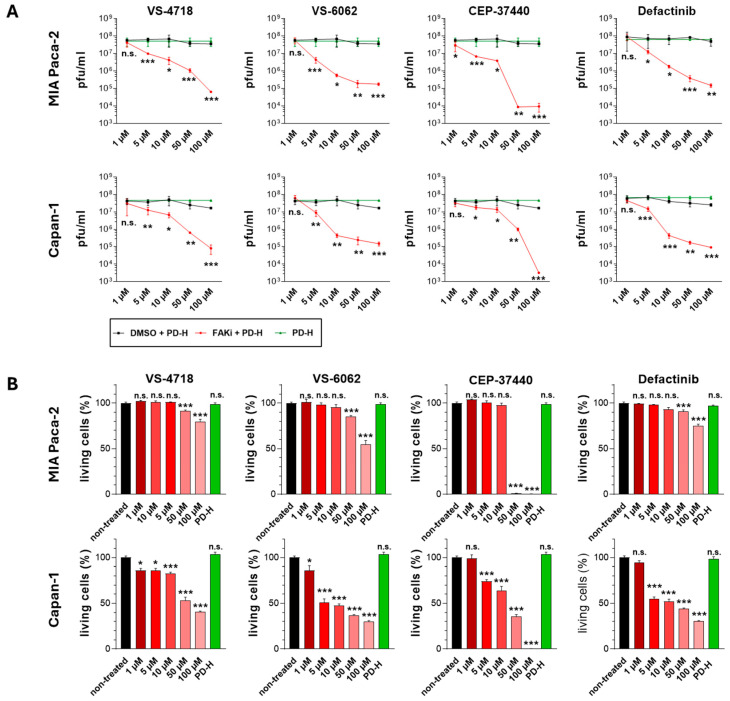
Influence of FAKi on the replication and cytotoxicity of PD-H. (**A**) Viral growth curves. MIA Paca-2 and Capan-1 cells were infected with 3 and 0.1 MOI of PD-H for 1 h and thereafter incubated with medium containing the FAKi VS-4718, VS-6062, CEP-37440, or Defactinib at indicated concentrations, or DMSO, or cell culture medium alone for 24 h. Virus titers were determined by plaque assay on HeLa cells. Shown are mean values ± SD from 3 independent experiments. Statistical significance compared to PD-H + DMSO, * *p* < 0.05, ** *p* < 0.01, and *** *p* < 0.001; n.s., not significant. (**B**) Cell viability of the PD-H/FAKi approach described under (**A**). Cell viability was determined by XTT assay 24 h post-treatment. Data were set relative to DMSO-treated cells. Statistical significance compared to non-treated cells, * *p* < 0.05 and *** *p* < 0.001; n.s., not significant.

The occurrence of antagonistic interactions of FAKi and PD-H and the lack of increased cytotoxicity with various FAKi/PD-H combination therapies raise the question of potential underlying causes. Since the FAKi are highly cytotoxic, we hypothesized that they might inhibit the replication of PD-H, thereby diminishing the beneficial effects of combining both agents. To this end, we examined viral replication in the cell lines MIA Paca-2 and Capan-1, which showed markedly different responses to the FAKi/PD-H combination, in the presence and absence of FAKi. As MIA Paca-2 cells are distinctly more sensitive than Capan-1 cells, they were treated with 3 MOI, whereas Capan-1 cells were treated with 0.1 MOI of PD-H. Both cell lines were co-treated with VS-4718, VS-6062, CEP-37440, or Defactinib at concentrations ranging from 1 µM to 100 µM, and viral replication was analyzed by plaque assay after 24 h. All FAKi led to dose-dependent inhibition of the replication of PD-H (Figure 6A). The most pronounced inhibition of PD-H replication was caused by CEP-37440, with a 5300-fold reduction of viral replication in Capan-1 at 100 µM, and a 3800–4300-fold decrease in MIA Paca-2 at 50 µM and 100 µM. At 10 µM and 5 µM, reductions in the replication of PD-H were more moderate (4-/18-fold and 2-/9-fold, respectively) and negligible at 1 µM in both cell lines. Strong inhibition of viral replication was also found for the other FAKi. Treatment with 100 µM VS-4718 reduced PD-H replication by 207-fold in Capan-1 and 554-fold in MIA Paca-2. At lower concentrations, inhibition diminished progressively—39-/35-fold (50 µM), 7-/16-fold (10 µM), and 3-/6-fold (5 µM)—with no inhibition at 1 µM in Capan-1 and MIA Paca-2 cells, respectively. VS-6062 led to strong suppression of PD-H replication in Capan-1 and MIA Paca-2 cells at 100 µM (113- and 200-fold), with decreasing effects at 50 µM (102-/190-fold), 10 µM (104-/120-fold), and 5 µM (4-/14-fold), and no inhibition at 1 µM. Similarly, Defactinib reduced replication by 280-fold (Capan-1) and 327-fold (MIA Paca-2) at 100 µM. Inhibition decreased with lower FAKi concentrations: 183-/214-fold (50 µM), 91-/39-fold (10 µM), 5-/6-fold (5 µM), and was absent at 1 µM. To gain a deeper understanding of the mechanisms underlying the inhibition of viral replication by FAKi, we sought to determine whether the observed reduction in viral replication is solely attributable to FAKi-induced cytotoxicity or whether FAKi could also directly affect viral replication. To address this, we assessed the cell viability in both cell lines 24 h after treatment (Figure 6B). In MIA Paca-2 cells, substantial cytotoxic effects were observed only at high FAKi doses of 100 µM and 50 µM, although the extent varied depending on the specific FAKi used. No cytotoxicity was detected at concentrations between 10 µM and 1 µM, regardless of the FAKi administered. In Capan-1 cells, a dose-dependent significant reduction in cell viability was observed at concentrations of 100 µM to 1 µM of VS-4718 and VS-6062 and from 100 µM to 5 µM for CEP-37440 and Defactinib. However, at the lowest tested concentration of 1 µM, CEP-37440 and Defactinib did not significantly impair cell viability (Figure 6B).

In summary, the data show that all investigated FAKi significantly inhibit PD-H replication in both cell lines. Mechanistically, this inhibition is closely related to FAKi-induced cytotoxicity, as evidenced by the substantial reduction in viral replication (2–3 log_10_) at FAKi concentrations which induced strong cytotoxicity. However, FAKi also appears to directly inhibit viral replication. This is illustrated by the significant reduction in PD-H replication at medium to low (MIA Paca-2) and low (Capan-1) FAKi concentrations, at which no cell cytotoxicity was observed. The latter is supported by previous findings in other viral models; for example, FAK inhibition has been shown to reduce replication of influenza A virus [45].

## 3. Discussion

Pancreatic cancer remains one of the most difficult malignancies to treat, with poor prognosis and low survival rates. This is largely due to the limited availability of effective therapies and the slow integration of new treatments into clinical practice. The development of novel targeted treatment approaches, such as small-molecule FAKi is therefore of high interest [16]. Given the central role of FAK in cancer progression and encouraging preclinical data supporting its therapeutic potential, several FAKi are currently under clinical evaluation across various cancer types, including PDAC. However, despite promising preclinical results, monotherapy with FAKi has shown only modest efficacy in clinical settings [22,23,24,46].

In this study, we systematically evaluated the cytotoxic efficacy of six clinical-grade FAKi across a panel of pancreatic tumor cell lines. Our findings demonstrate that most of these FAKi effectively reduce cell viability in vitro, with CEP-37440 and Defactinib being the most potent at low micromolar concentrations. Slightly less effective were VS-4718, Ifebemtinib, and VS-6062. In contrast, GSK2256098 displayed only low cytotoxic activity. The latter was a surprising result, as a previous study revealed that GSK2256098 has potent oncolytic activity in pancreatic cancer cells [47]. Since attenuation of FAK autophosphorylation at Tyr-397 is the primary mechanism of action of all FAKi tested, including GSK2256098, it can be ruled out that the differences we observed in the oncolytic activity of FAKi are due to a different mode of action in inhibiting FAK. It is more likely that differences in the molecule structure of the FAKi lead to variations in their pharmacokinetics, which contribute to different activities in the tumor cell lines. However, other mechanisms cannot be excluded. For example, VS-4718, VS-6062, and Defactinib additionally inhibit the PYK2 [19], and CEP-37440 targets the anaplastic lymphoma kinase (ALK) [48]. Both kinases are involved in tumor cell proliferation in pancreatic cancer [49,50], suggesting that their inhibition can further enhance the oncolytic activity of these FAKi.

Our investigations also revealed differential sensitivity of the analyzed pancreatic tumor cell lines to FAKi treatment, with Capan-1 and Beta-TC-3 cells being highly responsive to FAKi treatment and MIA Paca-2 displaying general low responsiveness. This finding corroborates previous observations by Zhang et al., who also reported variable sensitivity of pancreatic tumor cells to FAKi treatment [47]. Moreover, it highlights challenges related to FAKi therapy in patients with pancreatic cancer as PDACs of certain patients may likewise exhibit resistance to FAKi. Early identification of patients unlikely to benefit from FAKi therapy could prevent unnecessary treatment and minimize the risk of therapeutic failure. This could be achieved, for example, by using predictive biomarkers or by assessing FAKi efficacy in patient-derived tumor specimens. Such strategies may ultimately allow for a more precise and promising application of FAKi therapy.

Another aspect of our study was the evaluation of the combinatorial potential of FAKi with the oncolytic CVB3 PD-H. Indeed, some combinations, particularly VS-4718/PD-H and CEP-37440/PD-H resulted in enhanced cytotoxicity compared to either agent alone, with additive and even synergistic interactions in several cell lines calculated by the use of the mathematical prediction model of Chou and Talalay. The effect was most pronounced in Capan-1, AsPC-1, and Beta-TC-3 cells, whereas in MIA Paca-2 and BxPC-3 cells, the combination of FAKi and PD-H exhibited little to no enhancement of oncolytic activity, and frequently antagonistic interactions between FAKi and PD-H were observed. As a key mechanism underlying the different outcomes of the combination approach, we identified the inhibition of viral replication by FAKi. All tested FAKi suppressed PD-H replication in a dose-dependent manner, even at subcytotoxic concentrations. This effect was consistent in both FAKi-sensitive (Capan-1) and FAKi-resistant (MIA Paca-2) cell lines, indicating that FAKi per se disrupt viral replication machinery irrespective of cell line sensitivity. However, in MIA Paca-2 cells, very high FAKi concentrations were required to induce cytotoxicity. At these doses, viral replication is severely impaired, and PD-H may therefore be unable to exert its activity, leading to a lack of enhancement in cytotoxic efficacy within the combination approach. In contrast, in Capan-1 cells, low concentrations of FAKi were sufficient to elicit a pronounced cytotoxic effect, while viral replication remains largely unaffected under these conditions. Consequently, simultaneous treatment of Capan-1 cells with FAKi at low concentration and PD-H leads to an increase in cytotoxicity. These results are important for the clinical use of FAKi/OV combination therapies, as they indicate the occurrence of responders and non-responders when using the FAKi/OV combination. In order to keep the proportion of non-responders as low as possible, optimization of the combination strategy appears necessary. This includes the selection of FAKi that have the highest therapeutic efficacy in combination with OV, the use of highly replicative OV capable of overcoming FAKi-induced inhibition, and the implementation of rationally designed treatment regimens—such as sequential rather than simultaneous administration of FAKi and OV. Such refinements may help to reduce treatment failure and maximize therapeutic benefit.

The results of our study provide a foundation for further investigation into the therapeutic potential of combining FAKi with PD-H in pancreatic cancer. Based on the promising findings presented here, we are currently conducting in vivo studies to evaluate the therapeutic efficacy of the PD-H and FAKi VS-4718 combination in a pancreatic cancer model. Moreover, the incorporation of additional therapeutic strategies, such as immune checkpoint inhibitors, into the FAKi/OV combination regimen may further augment anti-tumor efficacy and therefore warrants investigation in future studies.

In summary, our study shows that various FAKi currently under clinical investigations for the treatment of different types of cancer are capable of killing pancreatic cancer cells. Furthermore, their oncolytic efficacy can be significantly improved by combining with the oncolytic CVB3 PD-H. The combination with PD-H, therefore, has the potential to improve the efficacy of FAKi treatment in pancreatic cancer.

## 4. Materials and Methods

### 4.1. Cell Culture

The Beta-TC-3 (murine pancreatic insulinoma beta-tumor) and MIA Paca-2 (human pancreatic cancer cell line) cells were cultured in high glucose Dulbecco’s modified Eagle’s medium (DMEM; Biowest, Darmstadt, Germany) with 10% fetal calf serum (FCS; c.c. pro, Oberdorla, Germany), 1% L-glutamine (Merck KGaA, Darmstadt, Germany), and 1% each of penicillin and streptomycin (P/S; AppliChem, Darmstadt, Germany). The AsPC-1, Capan-1, and BxPC-3 cell lines (human pancreatic cancer cell lines) were grown in RPMI 1640 (c.c.pro) supplemented with 10% FCS, 1% P/S, and 1% L-glutamine. The Beta-TC-3 and BxPC-3 cell lines were obtained from the Leibniz Institute DSMZ (German Collection of Microorganisms and Cell Cultures GmbH, Braunschweig, Germany). The AsPC-1, Capan-1 and MIA Paca-2 cell lines were purchased from CLS (Cell Lines Service GmbH, Eppelheim, Germany). HeLa cells were cultured in minimum essential medium (MEM; Gibco, Thermo Fisher Scientific Inc., Waltham, MA, USA) supplemented with 5% FCS, 0.02 M 2-[4-(2-hydroxyethyl)piperazin-1-yl]ethanesulfonic acid (HEPES; Gibco, Thermo Fisher Scientific Inc.), 1% non-essential amino acids (NEAA; Gibco, Thermo Fisher Scientific Inc.), and 1% P/S.

### 4.2. Virus

The oncolytic virus CVB3 PD-H was produced as described previously [38]. Briefly, to generate PD-H, the plasmid pJET-CVB3-PD-H containing the cDNA of the CVB3 strain PD-H was linearized with *Pme*I. Purified plasmid DNA was transcribed using the in vitro T7 Transcription Kit (Roboklon GmbH, Berlin, Germany). The RNA was transfected in CHO-K1 cells using PEImax (Polysciences Europe GmbH, Hirschberg an der Bergstraße, Germany). For further amplification of PD-H, CHO-K1 cells were infected with 0.3 MOI of PD-H for 48 h. For virus release, infected cells were subjected to three freeze and thaw cycles. The cell debris was removed by centrifugation, and the viral titer of the supernatant was analyzed by plaque assay on HeLa cells.

### 4.3. FAKi

The Inhibitors of the FAK VS-4718 (PND-1186), Ifebemtinib (BI-853520), VS-6062 (PF-00562271), GSK2256098, CEP-37440, and Defactinib (VS-6063) were purchased as 100% DMSO dissolved solution (10 mM) from MedChemExpress (Sollentuna, Sweden) and stored as aliquots at −80 °C up to use.

### 4.4. Cell Viability Assay

Cell viability was measured using the Cell Proliferation Kit (XTT; Promega GmbH, Walldorf, Germany) according to the instructions of the manufacturer. Cells were seeded in 96-well plates to reach approximately 80% confluence the following day. Cells were either treated with FAKi, infected with PD-H or both. After the incubation period, absorbance levels were measured using the TriStar5 LB 942 Multimode Microplate Reader (Berthold Technologies, Bad Wildbad, Germany). As a control for cell death, cells were treated with 50 µL 5% Triton X-100 solution (Carl Roth GmbH + Co. KG, Karlsruhe, Germany).

### 4.5. Determination of IC_50_ of FAKi

Cells were seeded in 96-well plates to reach approximately 80% confluence the following day. Cell medium was changed by fresh cell culture medium alone or containing FAKi (VS-4718, Ifebemtinib, VS-6062, GSK2256098, CEP-37440 or Defactinib) at concentrations of 0.1–100/500 µM, or DMSO in a corresponding solution. Cell viability was measured by XTT assay after 72 h. For each FAKi, a nonlinear fit was performed in GraphPad Prism (Version 8.4.3 GraphPad Software, Boston, MA, USA) and the IC_50_ value for each FAKi and for each cell line was calculated by interpolating the X value.

### 4.6. Determination of IC_50_ of PD-H

Cells were seeded in 96-well plates to reach approximately 80% confluence the following day. The cells were then infected with PD-H at concentrations of 0.001 to 50 MOI at 37 °C for 1 h in serum-free medium. Cells that were not infected (control) were only incubated with serum-free medium. After the incubation period, the medium was replaced with fresh cell culture medium. Cell viability was measured 72 h or 48 h (for Beta-TC-3 cells) post-infection by XTT assay. The determination of the IC_50_ values of PD-H for each cell line was carried out as described under Section 4.5.

### 4.7. Determination of the Combination Index Using the Chou–Talalay Constant Ratio Approach

Cells were seeded in 96-well plates to reach approximately 80% confluence the following day. Cells were infected with PD-H in serum-free medium or treated with serum-free medium alone for 1 h. Thereafter, the medium was replaced with fresh medium containing the FAKi, DMSO, or medium alone. The combination of FAKi with PD-H was applied according to the constant ratio approach as described earlier [41,44]. XTT assay was measured 72 h or 48 h (for Beta-TC-3) post-treatment. The data obtained were used to calculate the combination index (CI) for each combination approach using the CompuSyn software (version 1.0, ComboSyn, Inc., Paramus, NJ, USA) [51].

### 4.8. Virus Growth Curves

MIA Paca-2 and Capan-1 cells were seeded into 96-well plates to reach approximately 80% confluence the following day. For infection, the medium was replaced by serum-free medium containing 3 or 0.1 MOI of PD-H. After incubating for 1 h at 37 °C, the virus suspension was replaced by fresh cell culture medium, by medium containing the FAKi at concentrations from 1–100 µM, or DMSO in corresponding dilutions. Cell viability was measured 24 h post-infections. After the XTT assay, cells were frozen, followed by lysis through three freeze–thaw cycles. The cell debris was discarded by centrifugation, and the virus titers from the supernatant were analyzed by plaque assay.

### 4.9. Virus Plaque Assay

Plaque assays were carried out on HeLa cells as described previously [41].

### 4.10. Western Blot

Protein extraction was carried out using the general lysis buffer (20 mmol/L TRIS (Trishydroxymethylaminomethane), pH 8.0, 10 mmol/L NaCl, 0.5% Triton X-100, 5 mmol/L EDTA, and 3 mmol/L MgCl_2_) containing a protease and phosphatase inhibitor mixture (Merck KGaA, Darmstadt, Germany). Protein concentration was determined using the BCA Protein Assay Kit (Thermo Fisher Scientific Inc.). Protein samples (40 µg) were electrophoretically separated on 4–12% polyacrylamide gels (NuPAGE Bis-TRIS Gels; Thermo Fisher Scientific Inc.) and transferred to a polyvinylidene difluoride membrane (PVDF Western Blotting Membranes; Merck KGaA). The membranes were blocked with blocking buffer (5% milk in TRIS-buffered saline plus 0.1% Tween-20) for 1 h at room temperature. To detect P_397_-FAK (Tyr397-p125FAK) proteins, the Phospho-FAK (Tyr397) rabbit monoclonal antibody (31H5L17) (1:500; Thermo Fisher Scientific Inc.) and to detect total FAK proteins (p125FAK), the FAK mouse monoclonal antibody (1:500; clone 4.47 Merck KGaA) were applied for 24 h at 4 °C. As secondary antibodies, the horseradish peroxidase conjugated antibody was used (1:2000), 1 h at room temperature; highly cross-absorbed, Thermo Fisher Scientific Inc.). β-actin was used as the internal-loading control (mouse monoclonal antibody 8H10D10 (1:5000), 1 h at room temperature, Cell Signaling Technology, Inc., WZ Leiden, The Netherlands). Membranes were washed with TRIS-buffered saline plus 0.1% Tween-20. Images were taken using the Bio-Rad Chemidoc^TM^ MP Imaging System with Image Lab Software version 5.0 (Bio-Rad Laboratories GmbH, Feldkirchen, Germany). Quantification of the expression of proteins was carried out relative to the expression of β-actin by densitometric analysis using ImageJ version 1.53a densitometry software.

### 4.11. Statistical Analysis

Statistical significance was determined by an unpaired Student’s *t*-test. A *p*-value of <0.05 was considered statistically significant.

## Figures and Tables

**Table 1:** FAKi used in this study, targeted cancer type, identification number and phase of the clinical trial, molecular target, and structural formula.

FAKi	Clinical Trial/Phase		Targeted Kinases	Structural Formula
VS-4718 (PND-1186)	Acute Myeloid Leukemia, non-hematologic cancers, metastatic cancer, advanced PDAC	NCT01849744: I NCT02215629: I NCT02651727: I	FAK PYK2	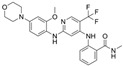
Ifebemtinib (BI-853520; IN-10018)	non-hematologic malignancies, various advanced or metastatic solid tumors	16 in sum: I (5), I/II (9), II (2)	FAK FER/FES	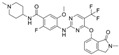
VS-6062 (PF-00562271)	advanced solid tumors	NCT00666926: I	FAK PYK2	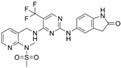
GSK2256098	PDAC, advanced solid tumors, progressive meningiomas	NCT02428270: II NCT00996671: I NCT02551653: I NCT01938443: I NCT01138033: I NCT02523014: II	FAK	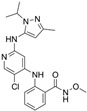
CEP-37440	advanced or metastatic tumors	NCT01922752: I	FAK ALK	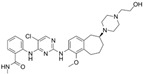
Defactinib (VS-6063; PF-04554878)	advanced solid cancer, lung cancer, non-hematologic cancers, ovarian cancer, glioblastoma, mesothelioma, PDAC	36 in sum: I (11), I/II (6), II (18), III (1)	FAK PYK2	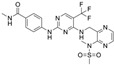

FAK, focal adhesion kinase; FER, FER tyrosine kinase; FES, FES tyrosine kinase; PYK2, tyrosine kinase 2 beta; ALK, anaplastic lymphoma kinase; from www.clinicaltrials.gov (accessed on 11 July 2025); chemical structural formula from www.medchemexpress.com (accessed on 11 July 2025).

**Table 2:** IC_50_ values of FAKi in pancreatic tumor cell lines.

Pancreatic Tumor Cell Line	IC_50_ [µM] VS-4718	IC_50_ [µM] Ifebemtinib	IC_50_ [µM] VS-6062	IC_50_ [µM] GSK2256098	IC_50_ [µM] CEP-37440	IC_50_ [µM] Defactinib
MIA Paca-2	60.85	36.99	44.29	422.95	23.30	20.26
BxPC-3	25.83	22.47	5.09	222.16	10.57	5.66
Capan-1	6.53	24.15	3.66	123.78	6.52	4.17
AsPC-1	12.47	27.47	15.64	740.58	9.48	12.34
Beta-TC-3	3.69	18.65	31.67	35.36	6.28	9.30
**Average IC_50_ [µM]**	**21.87**	**25.95**	**20.07**	**308.97**	**11.23**	**10.35**

The IC_50_ values of each FAKi were calculated from the measurements of FAKi-induced cytotoxicity shown in Figure 2. The average FAKi IC_50_ was calculated from the IC_50_ values determined for the FAKi in the five pancreatic tumor cell lines.

## Data Availability

Data supporting the results of this study are available from the corresponding author upon reasonable request.
